# Supplementary material for: Robotized indoor phenotyping allows genomic prediction of adaptive traits in the field
Source: Nat Commun. 2023 Oct 19;14:6603. doi: 10.1038/s41467-023-42298-z (PMC10587076; doi:10.1038/s41467-023-42298-z)
Supplement: Supplementary file 1 — Supplementary Information [file 41467_2023_42298_MOESM1_ESM.pdf]

Robotized indoor phenotyping allows genomic prediction of adaptive traits in the field

Bouidghaghen *et al.*

Supplementary Table 1. Panels of hybrids and corresponding experiments with analysed traits in this study.

Panel	Hybrid number	Composition	Indoor experiment number	Used traits in indoor experiments	Field experiment number	Used traits in field experiments	More info in
Diversity	246	246 dent lines x flint line UH007	4	LAR, LER, gs, rh_{PAD}, Leaf dimensions	25	Duration of vegetative phase, Leaf dimensions	https://doi.org/10.1038/s41588-019-0414-y
Genetic progress	56	Successful hybrids released from 1950 to 2015	4	LAR, LER, gs, rh_{PAD}, LER sensitivity to VPD & SWP, Final leaf number	26	Duration of vegetative phase, ALA, LAI	https://doi.org/10.15454/KLD0GH
Recent hybrids	86	Recent hybrids released from 2008 to 2020	1*	LAR, LER, rh_{PAD}	4	Duration of vegetative phase, LAR, ALA	Supplementary Tables 2 and 3, https://data.inra.fr/dataset.xhtml?persistentId=doi:10.15454/IASSTN

*One indoor platform experiment with a subset of 20 recent hybrids. LAR: Leaf Appearance Rate. LER: Leaf Expansion Rate. gs: Stomatal Conductance. rh_{PAD}: relative height at 50% of leaf area. Leaf dimensions: length and width. VPD: Vapour-pressure deficit. ALA: Average Leaf inclination Angle. LAI: Leaf Area Index. Detailed information for hybrids and experiments used for the diversity panel and the genetic progress panel are available at the URLs provided in this table.

Supplementary Table 2. Experiments performed for the 'recent hybrids' panel and experiments carried out at Field 5, Field 6 & Indoor for 'genetic progress' panel.

Panel	Name exp.	Type exp.	Location	GPS Coord.	Year	Treatment	# Hyb	Maturity groups	Measured traits
Recent hybrids panel	Field 1	Field	Matzenheim	48.401, 7.617	2022	WW	53	G2, G3	LAR, Emergence, Anthesis & Silking dates, Leaf 6 dimensions, Plant density, Final leaf number, Yield & its components
	Field 2	Field	Saint Bonnet de Mure	45.714, 5.046	2022	WW, WD	58	G3, G4	ALA, LAI, fiPAR, Emergence, Anthesis & Silking dates, Plant density, Final leaf number, Yield & its components
	Field 3	Field	Binas	47.919, 1.478	2022	WW	86	G2, G3, G4	LAR, Emergence, Anthesis & Silking dates, Leaf 6 dimensions, Plant density, Final leaf number, Yield & its components
	Field 4	Field	Pusignan	45.741, 5.075	2021	WW	54	G3, G4	ALA, LAI, fiPAR, Emergence, Plant density
	Indoor 1	Indoor Platform	Montpellier	43.618, 3.857	2021	WW, WD	20	G2, G3, G4	Leaf area, Biomass, LAR, LER, Plant architecture variables (rh_{PAD})
Genetic progress panel	Field 5	Field	Mauguio	43.611, 3.971	2017	WW, WD	56	G2, G3, G4	ALA, LAI, fiPAR, Emergence, Anthesis & Silking dates, Plant density, Final leaf number, Yield & its components
	Field 6	Field	Mauguio	43.610, 3.980	2010	WW, WD	44	G2, G3, G4	LAR, Emergence, Anthesis & Silking dates, Plant density, Final leaf number, Yield & its components
	Indoor 2	Indoor Platform	Montpellier	43.618, 3.857	2017	WW, WD	56	G2, G3, G4	Leaf area, Biomass, LAR, LER, gs, Plant architecture variables (rh_{PAD}), LER sensitivity to VPD & SWP, Final leaf number

Name exp.: Experiment name used in the text; Year, year when the experiment was done. Treatment: WW, imposed well-watered (controlled by sensors). WD, imposed water deficit (controlled by sensors)

Supplementary Table 3. Summary of variance components and genomic heritability estimated in the experiments considered in this study.

Traits	Unit	Experiments	# Hyb	Mean value	h_g^2	σ_g^2	σ_a^2	σ_d^2	σ_e^2
Leaf Appearance Rate (LAR)	leaf/ $day_{20°C}$	Indoor 2	44	0.275	0.64	1.9E-04	1.1E-04	8.0E-05	1.1E-04
		Field 6	44	0.281	0.61	7.7E-04	4.3E-04	3.3E-04	4.9E-04
		Indoor 1	21	0.271	0.58	2.7E-04	1.4E-04	1.3E-04	2.0E-04
		Field 3	26	0.260	0.53	1.5E-04	7.6E-05	7.2E-05	1.3E-04
		Field 5	44	0.287	0.64	4.6E-04	2.8E-04	1.8E-04	2.6E-04
		Field 1	26	0.262	0.53	1.3E-04	6.3E-05	7.1E-05	1.2E-04
Duration of the vegetative phase	$days_{20°C}$	Field 5	44	63.5	0.68	9.52	6.29	3.23	4.57
		Field 6	44	62.6	0.68	11.55	7.77	3.78	5.39
		Field 1	53	63.8	0.62	3.88	1.94	1.94	2.39
		Field 3	55	62.9	0.58	3.23	1.69	1.54	2.37
		Field 2	55	68.2	0.62	3.24	1.60	1.65	1.96
rh_{PAD} (relative height at 50% of leaf area) VS ALA (Average Leaf inclination Angle)	rh_{PAD} (unitless), ALA (°)	rh_{PAD} Indoor 2	56	0.277	0.69	1.3E-03	9.1E-04	4.4E-04	6.1E-04
		ALA Field 5	56	54.33	0.70	2.05	1.38	0.67	0.87
		rh_{PAD} Indoor 1	18	0.344	0.54	1.1E-03	5.8E-04	5.5E-04	9.5E-04
		ALA Field 4	18	67.24	0.56	0.84	0.43	0.40	0.67
		ALA Field 2	18	60.27	0.57	3.19	1.64	1.55	2.42
Leaf Area Index (LAI)		LAI Indoor 2 (+Crop model)	51	3.62	0.63	0.34	0.22	0.12	0.20
	unitless	LAI Field 5 (UAV)	51	3.67	0.68	0.11	0.07	0.04	0.05

#Hyb, number of hybrids. h_g^2, genomic heritability. σ_g^2, total genetic variance. σ_a^2 and σ_d^2, variances explained by additive and dominance relationship matrices, respectively. σ_e^2, residual variance.

Supplementary Table 4. Summary of correlation analysis and accuracy results estimated between experiments genotypic values for four traits.

Traits	Unit	Experiments	# Hyb	r	rho	Mean value	RMSE	CV_{RMSE}	Bias	CV_{Bias}	FSA to the highest quartile	FSA to the lowest quartile	iAcc
Leaf Appearance Rate (LAR)	leaf/ $day_{20°C}$	Indoor 2 VS Field 6	44	0.73 ± 0.07	0.68 ± 0.09	0.275	0.025	9.0%	0.006	2.1%	0.63	0.72	0.55 ± 0.11
		Indoor 1 VS Field 3	21	0.57 ± 0.16	0.42 ± 0.20	0.259	0.020	7.7%	-0.012	-4.5%	0.60	0.40	0.33 ± 0.21
		Field 5 VS Field 6	44	0.71 ± 0.08	0.43 ± 0.13	0.287	0.024	8.4%	-0.006	-2.0%	0.50	0.75	0.52 ± 0.11
		Field 1 VS Field 3	26	0.49 ± 0.16	0.35 ± 0.19	0.260	0.014	5.3%	-0.001	-0.3%	0.50	0.33	0.30 ± 0.19
Duration of the vegetative phase	$days_{20°C}$	Field 5 VS Field 6	44	0.88 ± 0.04	0.91 ± 0.03	63.5	2.2	3.5%	-0.95	-1.5%	0.81	0.72	0.63 ± 0.09
		Field 1 VS Field 3	53	0.69 ± 0.07	0.66 ± 0.09	61.7	2.7	4.4%	-1.94	-3.2%	0.54	0.62	0.47 ± 0.11
		Field 2 VS Field 3	55	0.47 ± 0.11	0.37 ± 0.12	64.1	4.5	7.0%	-4.05	-6.3%	0.60	0.37	0.31 ± 0.12
rh_{PAD} (relative height at 50% of leaf area) VS ALA (Average Leaf inclination Angle)	rh_{PAD} (unitless), ALA (°)	rh_{PAD} Indoor 2 VS ALA Field 5	56	0.77 ± 0.06	0.77 ± 0.06	0.28					0.71	0.57	0.55 ± 0.10
		rh_{PAD} Indoor 1 VS ALA Field 4	18	0.58 ± 0.17	0.51 ± 0.20	0.34	Irrelevant (two different traits compared)				0.60	0.60	0.33 ± 0.23
		rh_{PAD} Indoor 1 VS ALA Field 2	18	0.60 ± 0.17	0.60 ± 0.18	0.34					0.60	0.60	0.37 ± 0.22
		ALA Field 4 VS ALA Field 2	18	0.50 ± 0.19	0.42 ± 0.22	67.2	7.2	10.7%	-6.97	-10.4%	0.60	0.60	0.31 ± 0.23
Leaf Area Index (LAI)	unitless	LAI Indoor 2 (+Crop model) VS LAI Field 5_WW (UAV)	51	0.64 ± 0.09	0.66 ± 0.09	3.61	0.57	15.7%	0.06	1.7%	0.45	0.61	0.44 ± 0.12
		LAI Indoor 2 (+Crop model) VS LAI Field 5_WD (UAV)	51	0.44 ± 0.11	0.43 ± 0.12	2.74	0.54	19.6%	-0.20	-7.2%	0.33	0.50	0.31 ± 0.13

r, Pearson correlation coefficient. SE r, Standard Error of r. rho, Spearman correlation coefficient. SE rho, Standard Error of rho. e. RMSE, Root Mean Square Error. CV_{RMSE}, RMSE Coefficient of Variation. CV_{Bias}, Bias Coefficient of Variation. FSA to the highest quartile, Frequency of Similar Assignment to the highest quartile between experiments for each trait. FSA to the lowest quartile, Frequency of Similar Assignment to the lowest quartile between experiments for each trait. iAcc, Theoretical accuracy of indirect selection, i.e. in case of indirect selection based on trait observed values in a given experiment (indoor or in a field), calculated as the genetic correlation between the considered couple of experiments multiplied by the square root of trait genomic heritability in the reference experiment for selection[1]. Standard error (SE) estimates[2] are shown after the ± symbol.

Supplementary Table 5. Summary of G-BLUP genomic prediction results in cross-validation and external validation schemes.

Trait	Unit	5-fold Cross-Validation (Diversity and Genetic Progress panels)						External Validation (Recent hybrids panel)					
		# Hyb	Mean r	Mean rho	Mean RMSE	Observed mean value	Mean CV_{RMSE} (%)	# Hyb	r	rho	RMSE	Observed mean value	CV_{RMSE} (%)
Leaf Appearance Rate (LAR)	leaf/ $day_{20°C}$	302	0.58 ± 0.09	0.55 ± 0.10	0.013	0.254	5.2	50	0.53 ± 0.10	0.48 ± 0.12	0.007	0.262	2.8
Duration of the vegetative phase	$days_{20°C}$	302	0.84 ± 0.04	0.81 ± 0.05	1.8	67.3	2.7	60	0.71 ± 0.07	0.68 ± 0.08	1.6	64.6	2.5
rh_{PAD} (relative height at 50% of leaf area)	unitless	302	0.65 ± 0.08	0.57 ± 0.10	0.029	0.303	9.4	20	0.42 ± 0.20	0.35 ± 0.22	0.056	0.356	15.6
Stomatal conductance (gs_{max})	mmol/ m^2/s	302	0.56 ± 0.09	0.53 ± 0.10	9.30	110.40	8.4	Missing data					
Leaf Expansion Rate (LER)	cm^2/ $day_{20°C}$	302	0.76 ± 0.06	0.75 ± 0.06	12.17	140.12	8.7	20	0.34 ± 0.21	0.34 ± 0.22	20.75	146.91	14.1

Mean r, Mean rho, Mean RMSE, Mean CV_{RMSE}: Pearson & Spearman correlation coefficients, Root Mean Square Error and RMSE Coefficient of Variation, respectively, estimated between G-BLUP predicted values and measured values, averaged across folds and 10 iterations in a cross-validation scheme including diversity and genetic progress panels. r, rho, RMSE, CV_{RMSE}: Pearson & Spearman correlation coefficients, Root Mean Square Error and RMSE Coefficient of Variation, respectively, estimated between G-BLUP predicted values (with training on diversity and genetic progress panels) and measured values in recent hybrids panel, used as external validation. Standard error (SE) estimates[2] are shown after the ± symbol. Source data are provided as a Source Data file.

Supplementary Table 6. Summary of PC-BLUP model prediction results in cross-validation and external validation schemes.

Trait	Unit	5-fold Cross-Validation (Diversity and Genetic Progress panels)						External Validation (Recent hybrids panel)					
		# Hyb	Mean r	Mean rho	Mean RMSE	Observed mean value	Mean CV_{RMSE} (%)	# Hyb	r	rho	RMSE	Observed mean value	CV_{RMSE} (%)
Leaf Appearance Rate (LAR)	leaf/ $day_{20°C}$	302	0.53 ± 0.10	0.50 ± 0.11	0.014	0.254	5.4	50	0.11 ± 0.14	0.16 ± 0.14	0.011	0.262	4.1
Duration of the vegetative phase	$days_{20°C}$	302	0.72 ± 0.06	0.64 ± 0.09	2.3	67.3	3.4	60	0.11 ± 0.13	0.10 ± 0.13	2.7	64.6	4.2
rh_{PAD} (relative height at 50% of leaf area)	unitless	302	0.58 ± 0.09	0.45 ± 0.11	0.030	0.303	10.0	20	-0.21 ± 0.09	-0.19 ± 0.11	0.056	0.356	15.7
Stomatal conductance (gs_{max})	mmol/ m^2/s	302	0.41 ± 0.11	0.30 ± 0.12	10.09	110.40	9.1	Missing data					
Leaf Expansion Rate (LER)	cm^2/ $day_{20°C}$	302	0.61 ± 0.08	0.47 ± 0.11	15.26	140.12	10.9	20	0.25 ± 0.23	0.18 ± 0.24	17.80	146.91	12.1

Mean r, Mean rho, Mean RMSE, Mean CV_{RMSE}: Pearson & Spearman correlation coefficients, Root Mean Square Error and RMSE Coefficient of Variation, respectively, estimated between PC-BLUP predicted values and measured values, averaged across folds and 10 iterations in a cross-validation scheme including diversity and genetic progress panels. r, rho, RMSE, CV_{RMSE}: Pearson & Spearman correlation coefficients, Root Mean Square Error and RMSE Coefficient of Variation, respectively, estimated between PC-BLUP predicted values (with training on diversity and genetic progress panels) and measured values in recent hybrids panel, used as external validation. Standard error (SE) estimates[2] are shown after the ± symbol. Source data are provided as a Source Data file.

Supplementary Table 7. Diversity analysis of the studied panels using Nei's index of genetic diversity and observed heterozygosity level. Based on 440 000 polymorphic SNP markers.

	Nei's Genetic Diversity Index	Observed Heterozygoty
Diversity Panel (246 Parental Dent Lines)	0.45	0.00
Diversity Panel (246 Hybrids : Dent Lines x UH007)	0.31	0.44
Genetic Progress Panel (56 Hybrids)	0.36	0.38
Recent Hybrids Panel (86 Hybrids)	0.31	0.35

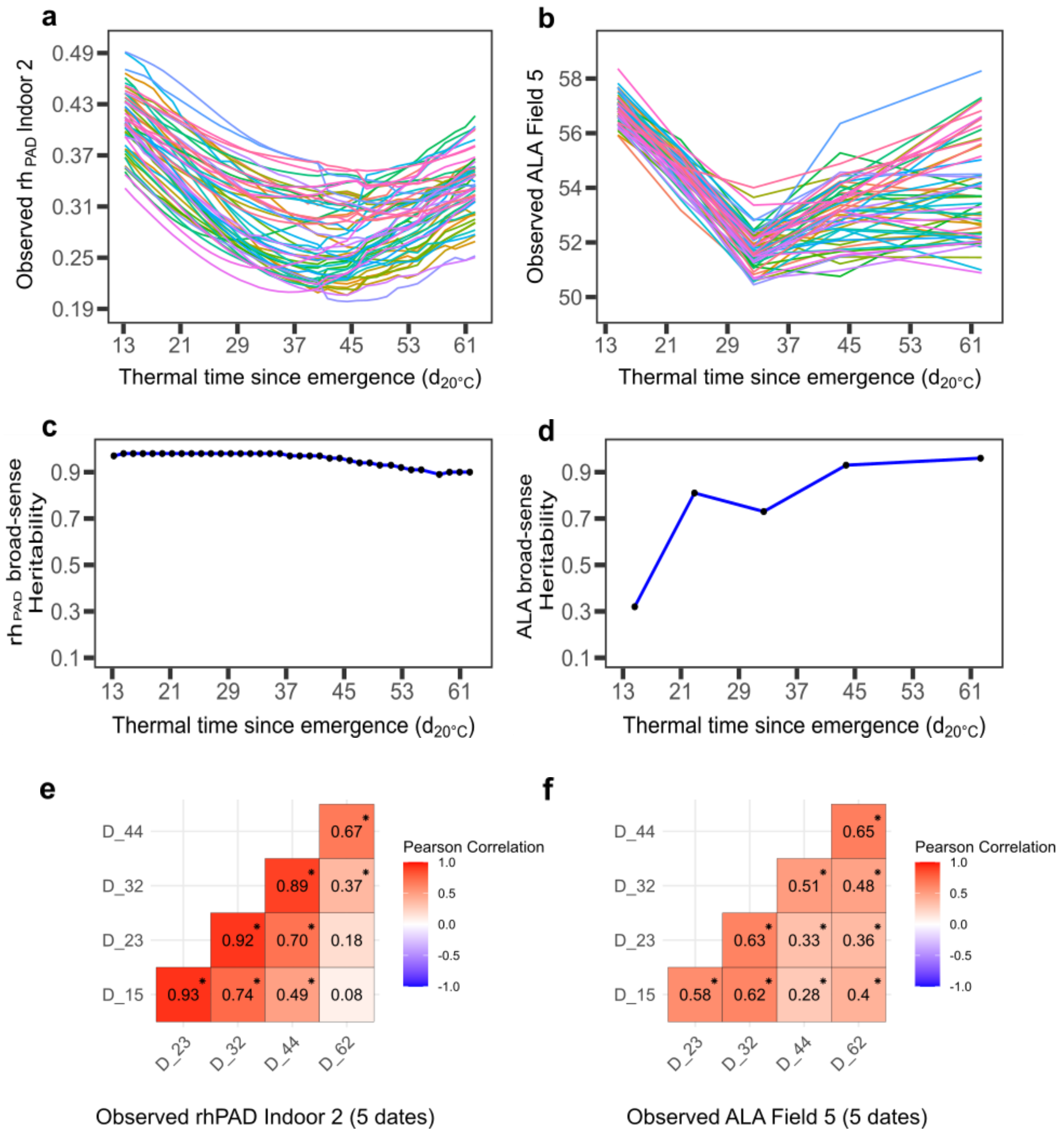

Supplementary Fig. 1. Time courses and broad-sense heritability over time of genotypic values of the architectural traits (rh_{PAD} and ALA) and their correlation heat maps for 5 dates. a & **b**, Time courses of rh_{PAD} observed in indoor plarform and ALA observed in the field until flowering. **c** & **d**, Broad-sense heritability over thermal time for rh_{PAD} and ALA. **e** & **f**, Heat map of Pearson correlations between 5 thermal time points for rh_{PAD} and ALA trait. rh_{PAD}, relative altitude, from the top of the plant, where 50% of leaf area is reached[3]. ALA: average leaf inclination angle estimated from UAV images, via inversion of the model PROSAIL[4,5]. In **a** & **b**, lines represent 56 different hybrids. In **e** & **f**, statistically significant correlations are shown with an asterisk. Source data are provided as a Source Data file.

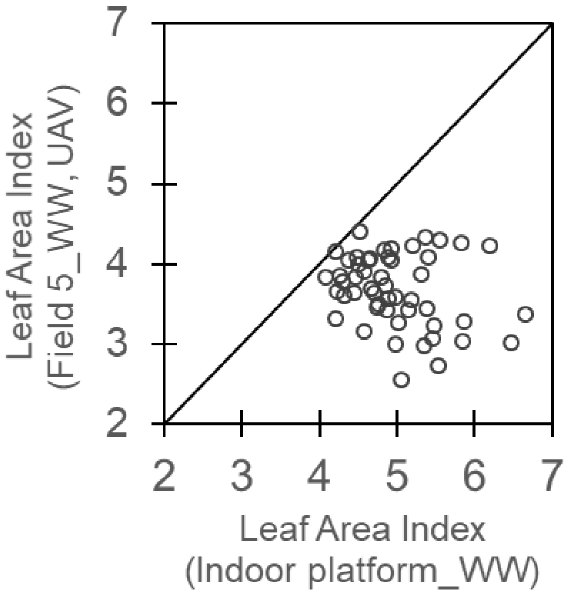

Supplementary Fig. 2. Genotypic values of leaf area index (LAI) measured in the field in well-watered condition were not correlated to LAI values calculated by considering only plant leaf area measured indoor.

Field values (on y-axis) were obtained at flowering time from UAV images via inversion of the model PROSAIL[4,6]. Values on x-axis were calculated as plant leaf area measured indoor at flowering time, multiplied by the plant density in the corresponding field. Each point represents a couple of observed genotypic values for one different hybrid of genetic progress panel. r = - 0.25, n=51, *p*-value = 0.073, CV_{RMSE} = 30.9%. Source data are provided as a Source Data file.

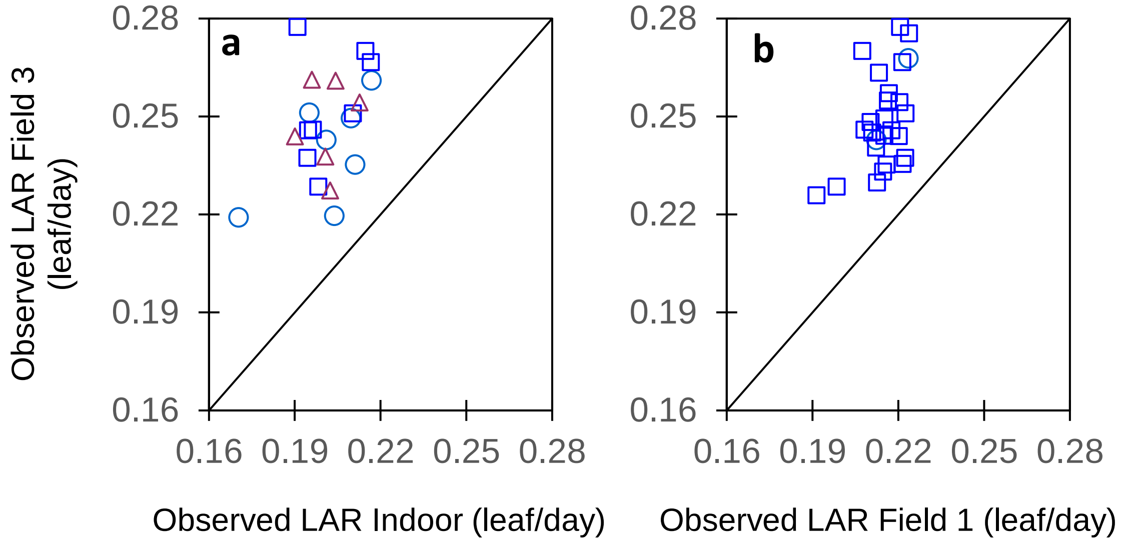

Supplementary Fig. 3. Leaf appearance rate (LAR) when calculated in calendar time had low correlations between genotypic values indoor and a field (a) or from one field to another field (b). Light blue circles, mid-early hybrids (G2), dark blue squares, intermediate hybrids (G3), red triangles, mid-late hybrids (G4). In **a**, r=0.40, n=21, *p*-value = 0.069, CV_{RMSE} = 23.8%. In **b**, r=0.49, n=26, *p*-value = 0.0117, CV_{RMSE} = 16.91%. Source data are provided as a Source Data file.

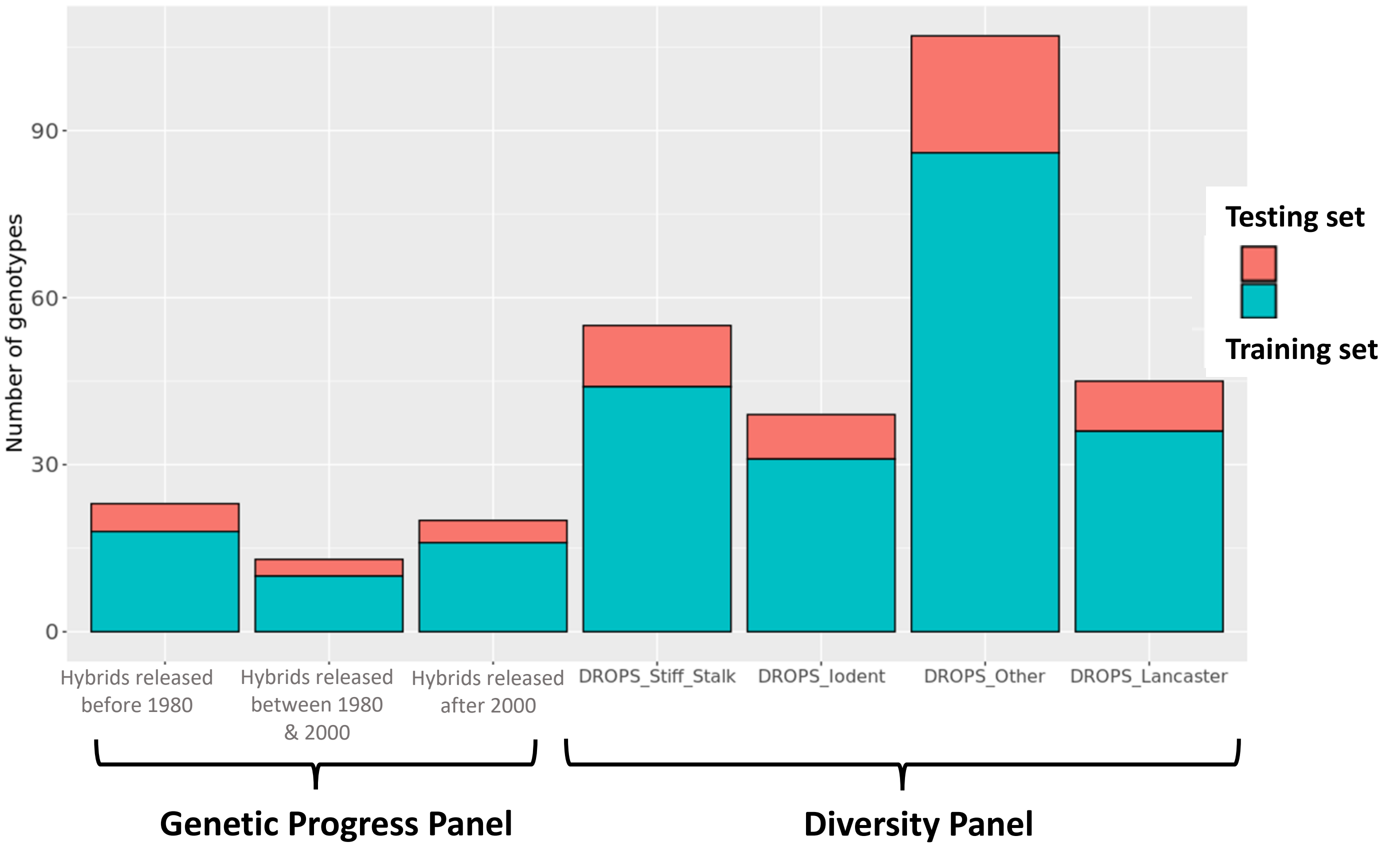

Supplementary Fig. 4. Schematic representation of genomic prediction cross-validation strategy. Each training set was sampled randomly but proportionally to diversity panel genetic groups and across years of release of genetic progress hybrids.

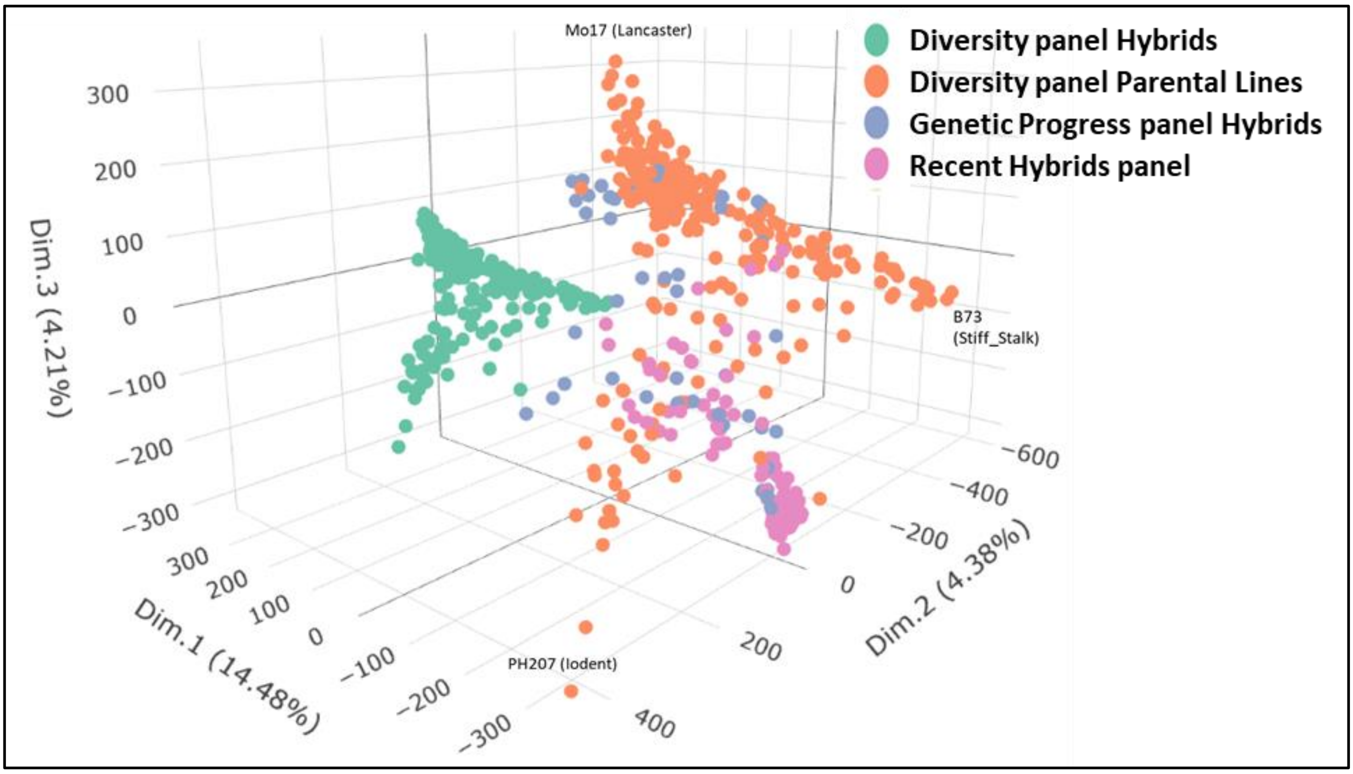

Supplementary Fig. 5. Structure and diversity analysis of the studied panels using a Principal Coordinate Analysis (PCoA) on SNP markers data. The PCoA was based on a set of 440 000 polymorphic SNP markers. The analysis first shows the structuration of the diversity panel parental lines into 4 genetic groups (Iodent on the bottom left, Lancaster on the top, Stiff_Stalk on the bottom right and the other diverse admixed genotypes in the middle). The genetic progress and recent hybrids panels mainly overlap with the Iodent and admixed genotypes of diversity panel. The representation of the diversity panel hybrids corresponding to the lines crossed with UH007, highlights the effect of the common parent UH007, which induces a strong genomic relatedness between the diversity panel hybrids and separates them from the other panels following the first PCoA dimension. Source data are provided as a Source Data file.

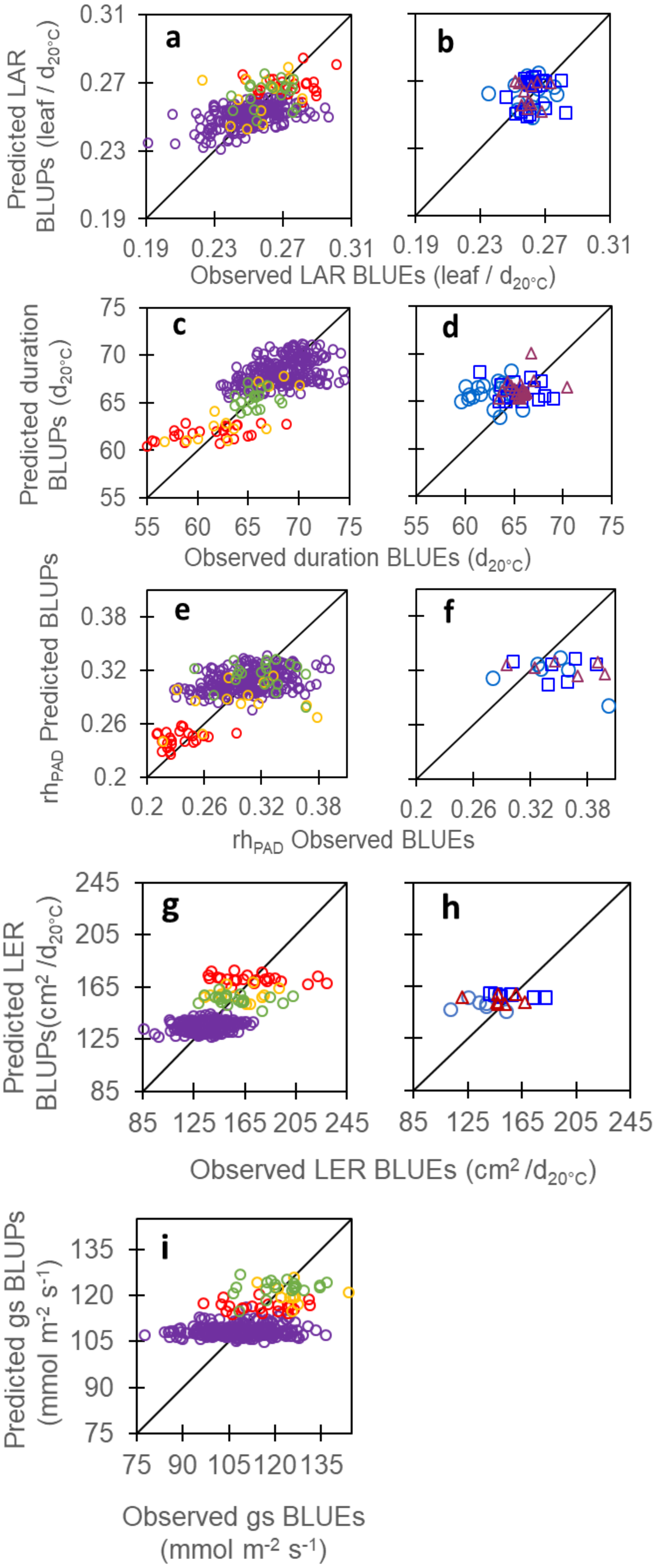
a
b
c
d
e
f
g
h
i
Predicted LAR BLUPs (leaf / d20°C)
Observed LAR BLUEs (leaf / d20°C)
Predicted duration BLUPs (d20°C)
Observed duration BLUEs (d20°C)
rhPAD Predicted BLUPs
rhPAD Observed BLUEs
Predicted LER BLUPs(cm2 /d20°C)
Observed LER BLUEs (cm2 /d20°C)
Predicted gs BLUPs (mmol m-2 s-1)
Observed gs BLUEs (mmol m-2 s-1)

Supplementary Fig. 6.The prediction of traits via a PC-BLUP model was less accurate than that with G-BLUP models in Fig. 1cd, 2cd, 3ef, 5b and 7ab for cross-validations (a, c, e, g, i), and lost accuracy for external validation (b, d, f, h). **a**, **c** & **e**, Comparison of observed mean genotypic values and mean predicted values in a 5-fold CV scheme with 10 iterations, in the 'diversity' and 'genetic progress' panels, for leaf appearance rate (LAR), duration of the vegetative phase & rh_{PAD} trait, respectively. **b**, **d** & **f**, Comparison of observed mean genotypic values and predicted values in an independent dataset of elite recent hybrids, with model calibration made using dataset of **a**, **c** & **e**, respectively. In **a**, **c** & **e**, purple empty circles, diversity panel; red and yellow empty circles, genetic progress panel, hybrids released before 1980 and 2000, respectively ; green empty circles, hybrids released after 2000. In **b**, **d** & **f**, light blue circles, mid-early hybrids (G2), dark blue squares, intermediate hybrids (G3), red triangles, mid-late hybrids (G4). In **a**, r = 0.53, n= 302, *p*-value < 2.2E-16, CV_{RMSE} = 5.4%. In **b**, r = 0.11, n= 50, *p*-value = 0.45, CV_{RMSE} = 4.1%. In **c**, r = 0.72, n= 302, *p*-value < 2.2E-16, CV_{RMSE} = 3.4%. In **d**, r = 0.11, n= 60, *p*-value = 0.39, CV_{RMSE} = 4.2%. In **e**, r = 0.58, n= 302, *p*-value < 2.2E-16, CV_{RMSE} = 10%. In **f**, r = -0.21, n= 20, *p*-value = 0.37, CV_{RMSE} = 15.7%. Source data are provided as a Source Data file.

Supplementary references

[1] Lopez-Cruz, M. *et al*. Regularized selection indices for breeding value prediction using hyper-spectral image data. *Sci Rep* **10**, 8195 (2020).

[2] Bonett, D. G. & Wright, T. A. Sample size requirements for estimating pearson, kendall and spearman correlations. *Psychometrika* **65**, 23–28 (2000).

[3] Perez, R. P. A. *et al*. Changes in the vertical distribution of leaf area enhanced light interception efficiency in maize over generations of selection. *Plant, Cell & Environment* **42**, 2105–2119 (2019).

[4] Berger, K. *et al*. Evaluation of the PROSAIL model capabilities for future hyperspectral model environments: A review study. *Remote Sensing* **10**, 85 (2018).

[5] Jiao, Q. *et al*. A random forest algorithm for retrieving canopy chlorophyll content of wheat and soybean trained with PROSAIL simulations using adjusted average leaf angle. *Remote Sensing* **14**, 98 (2021).

[6] Casa, R. *et al*. Estimation of maize canopy properties from remote sensing by inversion of 1-D and 4-D models. *Precision Agric* **11**, 319–334 (2010).
