# Supplementary material for: Robotized indoor phenotyping allows genomic prediction of adaptive traits in the field
Source: Nat Commun. 2023 Oct 19;14:6603. doi: 10.1038/s41467-023-42298-z (PMC10587076; doi:10.1038/s41467-023-42298-z)
Supplement: Supplementary file 3 — Description of Additional Supplementary information [file 41467_2023_42298_MOESM3_ESM.pdf]

Description of Additional Supplementary Files

File Name: Supplementary Data 1
Description: Hybrids of the 'recent hybrids' panel and experiments in which each of them was evaluated.
